# Coordinated evolution of brain size, structure, and eye size in Trinidadian killifish

**DOI:** 10.1002/ece3.7051

**Published:** 2020-11-22

**Authors:** Kaitlyn J. Howell, Shannon M. Beston, Sara Stearns, Matthew R. Walsh

**Affiliations:** ^1^ Department of Biology University of Texas at Arlington Arlington TX USA

**Keywords:** brain architecture, brain size, coordinated evolution, covariation, eye size, selection

## Abstract

Brain size, brain architecture, and eye size vary extensively in vertebrates. However, the extent to which the evolution of these components is intricately connected remains unclear. Trinidadian killifish, *Anablepsoides hartii*, are found in sites that differ in the presence and absence of large predatory fish. Decreased rates of predation are associated with evolutionary shifts in brain size; males from sites without predators have evolved a relatively larger brain and eye size than males from sites with predators. Here, we evaluated the extent to which the evolution of brain size, brain structure, and eye size covary in male killifish. We utilized wild‐caught and common garden‐reared specimens to determine whether specific components of the brain have evolved in response to differences in predation and to determine if there is covariation between the evolution of brain size, brain structure, and eye size. We observed consistent shifts in brain architecture in second generation common garden reared, but not wild caught preserved fish. Male killifish from sites that lack predators exhibited a significantly larger telencephalon, optic tectum, cerebellum, and dorsal medulla when compared with fish from sites with predators. We also found positive connections between the evolution of brain structure and eye size but not between overall brain size and eye size. These results provide evidence for evolutionary covariation between the components of the brain and eye size. Such results suggest that selection, directly or indirectly, acts upon specific regions of the brain, rather than overall brain size, to enhance visual capabilities.

## Graphical abstract

We evaluated the extent to which the evolution of brain size, brain structure, and eye size covary in male killifish from sites that vary in predation. We found positive connections between the evolution of brain structure and eye size but not between brain size and eye size. We also found that male killifish from sites that lack predators exhibited significantly larger brain structures than those from sites with predators. These results provide evidence for evolutionary covariation between the components of the brain and eye size and that selection, directly or indirectly, acts upon specific regions of the brain, rather than overall brain size, to enhance visual capabilities.

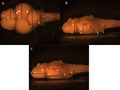

## 1 INTRODUCTION

Brain size and structure are well known to vary extensively across vertebrates (Andrew, 1962; Dunbar, 1998; Lefebvre et al., 2004; Striedter, 2005). A large body of research has shown that there is frequently a link between such variation and fitness. Increases in vertebrate brain size are positively correlated with a longer lifespan and increased survival (Amiel et al., 2011; González‐Lagos et al., 2010; Sol et al., 2007, 2008; Sol & Lefebvre, 2000). Large brains have also been associated with shifts in mating behavior, predator avoidance, learning, and behavioral flexibility (Buechel et al., 2018; Herczeg et al., 2019; A. Kotrschal et al., 2015; Ratcliffe et al., 2006; van der Bijl et al., 2015). Similarly, research has shown that individual components of the brain are correlated with shifts in cognitive abilities that may alter fitness (Garamszegi & Eens, 2004; Hutcheon et al., 2002; Lefebvre et al., 1997; Ratcliffe et al., 2006; Safi & Dechmann, 2005). For example, Hutcheon et al. (2002) showed that various brain structure sizes (i.e., auditory nuclei, olfactory bulb, hippocampus) were connected to foraging ecology in bats.

A growing body of literature has provided links between ecologically divergent conditions and evolutionary shifts in brain size. Ecological conditions such as habitat (Axelrod et al., 2018; Gonda, Herczeg, & Merilä, 2009a, 2011; Keagy et al., 2018; Park & Bell, 2010) and predation (Samuk et al., 2018; Walsh et al., 2016) have been linked to phenotypic shifts in brain size and brain structure. For example, Keagy et al. (2018) found that stickleback that are adapted to forage on benthic invertebrates ('benthic sticklebacks') exhibited larger relative brain volumes than stickleback that forage in open water environments (i.e., ‘limnetic sticklebacks'). This same study showed that benthic stickleback had larger relative optic tecta and smaller olfactory bulbs than limnetic fish (see also Park & Bell, 2010). Axelrod et al. (2018) found that brain size, but not structure, differed between sunfish from habitats that vary in structural complexity; sunfish from the more structurally complex, littoral habitats in a lake exhibited larger brains than those from open water. Samuk et al. (2018) performed a selection experiment in seminatural ponds and found that increased predation led to the evolution of significantly smaller brains and brain structures, specifically smaller optic lobes and telencephala, in sticklebacks. This growing body of work provides clear connections between divergent ecological conditions and shifts in brain size and brain architecture. A number of these studies have also shown that this phenotypic variation in brain size is likely genetically based as differences were maintained after multiple generations of laboratory common garden rearing (Gonda et al., 2009a; Samuk et al., 2018; Walsh et al., 2016).

In addition to variation in brain size and brain architecture, organisms also exhibit extensive variation in eye size (Land & Fernald, 1992). Increases in vertebrate eye size are associated with improved vision (Caves et al., 2017; Møller & Erritzøe, 2010; Motani et al., 1999; Walls, 1942) and thus enhanced fitness via shifts in foraging, predatory, and mating behavior (Garamszegi et al., 2002; Hall & Ross, 2007; Huber et al., 1997; Møller & Erritzøe, 2014; Thomas et al., 2006). Evolutionary shifts in eye size have also been linked to changes in ecological conditions. For example, studies have quantified selection on eye size due to such factors as light availability (Hall, 2008; Hiller‐Adams & Case, 1988; Kröger & Fernald, 1994; Veilleux & Lewis, 2011), predation (Glazier & Deptola, 2011; Møller & Erritzøe, 2010; Nilsson et al., 2012), and competition (Beston et al., 2017, 2019; Beston & Walsh, 2019). This growing body of work illustrating similar shifts in brain and eye size in response to ecologically mediated selection foreshadows the possibility that selection favors coordinated shifts in these neurosensory systems. However, the extent to which there are connections between the evolution of brain and eye size is unclear.

There are several reasons why the evolution of brain size, brain architecture, and eye size may be intricately linked. This is, in part, because there are well known connections between brain structures and organismal performance. For example, the telencephalon is linked to emotional learning, temporal and spatial memory, spatial cognition, and spatial behavior such as predator avoidance, foraging, and mating in teleost fish (Broglio et al., 2003; Portavella et al., 2002). The cerebellum is involved in controlling the execution of motor activity and is therefore important to memory and learning (Broglio et al., 2003). The medulla is involved with auditory function and relaying information between the brain and spinal cord (Collin et al., 2015; Tomchik & Lu, 2005). More importantly, various brain structures are connected to aspects of vision. Specifically, the vertebrate telencephalon receives visual information from the retina (Cooper et al., 1989; Garamszegi et al., 2002; Luiten, 1981), while the optic tectum is linked to multisensory integration, coordinated eye and body movements, and processes visual stimulus information (Broglio et al., 2003). Few studies have investigated potential covariation between overall brain size and eye size (Burton, 2008; Corral‐López et al., 2017; Garamszegi et al., 2002). These studies have found significant, positive associations between relative brain size and relative eye size across species of birds (Burton, 2008; Garamszegi et al., 2002). Artificial selection on brain size in guppies (*Poecilia reticulata*) showed that fish selected for larger brains also evolved larger eyes, but did not exhibit increased visual acuity (Corral‐López et al., 2017). However, tests of this connection across divergent ecological environments within a single species have yet to transpire.

Trinidadian killifish (historically *'Rivulus hartii*' but now *Anablepsoides hartii*) are found in sites that differ in predation intensity due to their ability to disperse and colonize novel upstream environments. This includes downstream, lowland 'high predation' (HP) sites where killifish co‐occur with several species of piscivorous fish (e.g., *Crenicichla frenata*, *Hoplias malabaricus*), and upstream, ‘*Rivulus*‐only’ (RO) sites where killifish are the only species of fish that is present (‘*Rivulus*‐only’ is used given historical precedent; Fraser et al., 1999; Gilliam et al., 1993; Walsh & Reznick, 2008). In HP sites, killifish experience increased mortality rates likely due to the presence of predators (Furness & Reznick, 2014). Several variables covary with the presence of predators that may also exert selection on the traits of killifish. HP sites exhibit a more open canopy cover (Reznick et al., 2001), the densities of killifish are lower, and per capita food availability is higher when compared to these same features in RO sites (Walsh & Reznick, 2008, 2009). Conversely, killifish are found at much higher densities in RO sites and thus experience strong intraspecific competition for limited resources (Gilliam et al., 1993; Walsh & Reznick, 2008). Research has shown that the ecological differences between HP and RO sites is associated with evolutionary divergence in life‐history traits (Walsh et al., 2011; Walsh & Reznick, 2008, 2009, 2010, 2011). Recent work has also revealed an association between increased predation and evolutionary shifts in brain and eye size between HP and RO sites (Beston et al., 2017; Walsh et al., 2016). Males, but not females, from RO sites have evolved larger brains than males from sites with predators. These trends were maintained after two generations of common garden rearing, which indicates that these differences are likely genetically based (Walsh et al., 2016). Similarly, male killifish from RO sites have also evolved a larger relative eye size than males from high predation sites (Beston & Walsh, 2019; Beston et al., 2017). These evolved differences in brain and eye size between sites with and without predators present the opportunity to determine whether increased predation has also driven shifts in brain architecture and whether there is a coordinated pattern of evolution among brain size, brain structure, and eye size.

Here, we evaluated the extent to which the evolution of brain size, brain structure, and eye size covary in killifish. We utilized wild‐caught and common garden‐reared specimens of killifish to address three specific questions. First, is there evidence that specific components of the brain have evolved in response to differences in predation? Second, are the patterns of brain architecture evolution consistent between wild‐caught and common garden‐reared fish? Lastly, is there covariation between the evolution of brain size, brain structure, and eye size? We predict that the telencephalon and optic tectum will be the strongest predictors of eye size, given that these structures are likely the most intricately linked to vision. That is, we expect to observe positive relationships between these brain structures and eye size.

## 2 MATERIALS AND METHODS

### 2.1 Wild caught specimens

We collected killifish from high predation and *Rivulus*‐only sites from the Arima, Aripo, and Guanapo rivers during May–June 2016 using small seines. All captured fish were immediately euthanized with MS‐222, preserved in 10% formalin, and then stored in 70% ethanol. Fish were measured for total length and photographed on their side for assessment of eye size using a Canon PowerShot ELPH180 or Nikon CoolPix S610 camera. Eyes were measured using the diameter of the eye cavity at the widest part for each photograph. All brains were then dissected during the Summer–Fall of 2016. Brains were dissected from each preserved specimen by cutting from the top of each gill slit and then removing the lower jaw and the tissue between the roof of the mouth and the braincase. All brains were stored in 70% ethanol until they were photographed for estimates of the volume of the brain structures in January 2019. To do so, we took separate images for the dorsal, lateral, and ventral views (Figure S1). All measurements were completed by individuals unaware of the population of origin. We then quantified the volume of the telencephalon, optic tecta, cerebellum, and dorsal medulla via the ellipsoid model: V = (L × W × H)*π*/6. This approach has been shown to provide a highly accurate estimate of the volume of fish brain structures (e.g. Huber et al., 1997; Pollen et al., 2007). Our total sample size included 143 male killifish (Arima high predation = 18 fish, Arima *Rivulus*‐only = 21, Aripo high predation = 24, Aripo *Rivulus*‐only = 13, Guanapo high predation = 25, Guanapo *Rivulus*‐only = 42).

We evaluated killifish from HP and RO sites for differences in the volume of the telencephalon, optic tectum, cerebellum, and dorsal medulla via linear mixed models (SPSS v. 26 IBM Corporation) with fish community (high predation, *Rivulus*‐only), river (Arima, Aripo, Guanapo), and all interactions entered as fixed effects. We ln‐transformed all traits to improve normality and homogeneity of variances and included ln‐fish length as a covariate. We first evaluated the full model with all possible interactions (including interactions with the covariate). We then removed interactions with little statistical significance (*F*‐value < 1.0) and reran analyses to converge on the best fitting model (lowest AIC values). We also performed complimentary analyses that included ln‐brain size as a covariate instead of body length. The results from both analyses were similar. In the results section, we focus on results that included total length as a covariate but also present the analyses using brain size as the covariate (see Table S1). Based upon the number of analyses performed for these data, we expected to observe approximately four significant results by chance.

### 2.2 Common garden specimens

Methods from the common garden experiments were previously published (Walsh & Reznick, 2008) and are briefly summarized here. We collected wild‐caught killifish from RO and HP sites in the Arima and Guanapo rivers in July 2005. We returned the fish to the laboratory and established laboratory stocks from approximately 20 fish per population (10 males and 10 females, 72 fish total). We generated the first laboratory generation by randomly pairing wild‐caught males and females from the same locality in 9‐l aquaria with an artificial spawning substrate. The eggs that were collected from each pairing were placed in Petri dishes in a methylene blue solution. All newly hatched larvae from each pairing were then placed in aquaria at a maximum density of eight fish per tank and were fed an ad libitum diet of liver paste and brine shrimp nauplii. We generated the second laboratory generation by pairing mature females from each lineage in the first generation with mature males from the same locality but different lineage. Overall, the experiment included fish from six pairings per population. For the common garden experiment, all offspring were reared at densities of eight fish per 9‐l aquarium and fed ad libitum. Beginning at an age of 20 days, eight fish from each pairing were individually placed in separate 9‐l aquaria. Each tank was supplied with a clay pot for refuge and an artificial spawning substrate. The fish from each pairing were equally divided between two food treatments: (a) a ‘high food’ ration that approximates growth in HP sites and (b) a low food ration that mimics the growth naturally observed in RO sites (Walsh & Reznick, 2008). All fish were provided with quantified portions of liver paste in the morning and brine shrimp nauplii in the afternoon. We then reared all killifish to maturity in order to quantify a diversity of life‐history traits (see Walsh & Reznick, 2008 for details regarding quantification of life‐history traits). Males were immediately euthanized and preserved in 5% formalin following maturation. All preserved fish were stored for approximately eight years prior to being photographed for eye size, weighed, and dissected for assessments of brain size and structure.

We dissected the brain from all preserved specimens beginning in August 2015 (see Walsh et al., 2016). The brain was removed by cutting from the top of each gill slit and then removing the lower jaw and the tissue between the roof of the mouth and the braincase. Each brain was blotted dry, and we then photographed the dorsal surface of each brain (Figure S1‐a). We then measured the width of the telencephalon, optic tecta, cerebellum, and dorsal medulla via ImageJ. All measurements were performed by individuals unaware of population of origin or food treatment. Note that we only have images associated with the dorsal surface of the brain and could therefore not calculate the volume of the brain structures. To address possible issues with comparing width (dorsal surface) to structure volume, we ran correlations between structure width and structure volume for each brain structure using wild caught data. All correlations were significant (*p* < .05, Table S2). The total sample size of common garden‐reared specimens was 87 males (Arima high predation = 22, Arima *Rivulus*‐only = 23, Guanapo high predation = 19, Guanapo *Rivulus*‐only = 23).

We evaluated killifish from HP and RO sites for differences in the width of the telencephalon, optic tectum, cerebellum, and dorsal medulla via linear mixed models (SPSS v. 26 IBM Corporation) with fish community (high predation, *Rivulus*‐only), food level (high, low), river (Arima, Guanapo), and all interactions entered as fixed effects. We ln‐transformed all traits to improve normality and homogeneity of variances and included ln‐fish length as a covariate. Similar to the analyses for the wild‐caught fish, we evaluated the full model with all possible interactions. We then removed interactions with little statistical significance (*F*‐value < 1.0) and reran analyses with reduced models to find the best fit model (lowest AIC values). Since the age of these fish was known (see Walsh & Reznick, 2008), we included their age at maturation as a covariate in the initial analyses. The influence of age at maturation was not significant for all traits. We therefore removed this covariate from the analyses. We also ran complimentary analyses that included ln‐brain size as a covariate instead of body length and found results were similar for both (see Table S3). Based upon the number of analyses performed for these data, we expected to observe approximately three significant results by chance.

### 2.3 Covariation between eye size and brain size

To further evaluate the extent to which the brain structures evolve in concert or independently with eye size, and also determine the structures that contribute to variation in brain and eye size, we first performed multiple regressions with ln‐transformed eye size as the dependent variable and each ln‐transformed brain structure and ln‐transformed brain size as the independent variables (SPSS v.26 IBM Corporation). Then, we separated the data by population and ran regressions to evaluate potential differences in the relationship between brain size, brain structure, and eye size across RO and HP sites. All regressions were performed with ln‐length as a covariate. To determine whether the connection between eye size and brain size differ between populations, we then specifically evaluated the ‘predation × brain’ interaction via general linear models with ln‐eye size as the dependent variable, predation as a fixed effect, and ln‐brain size or brain structure as a covariate. All of these analyses were performed separately for wild caught and common garden‐reared fish.

## 3 RESULTS

### 3.1 Brain structure

#### 3.1.1 Wild caught

**Figure 1:**
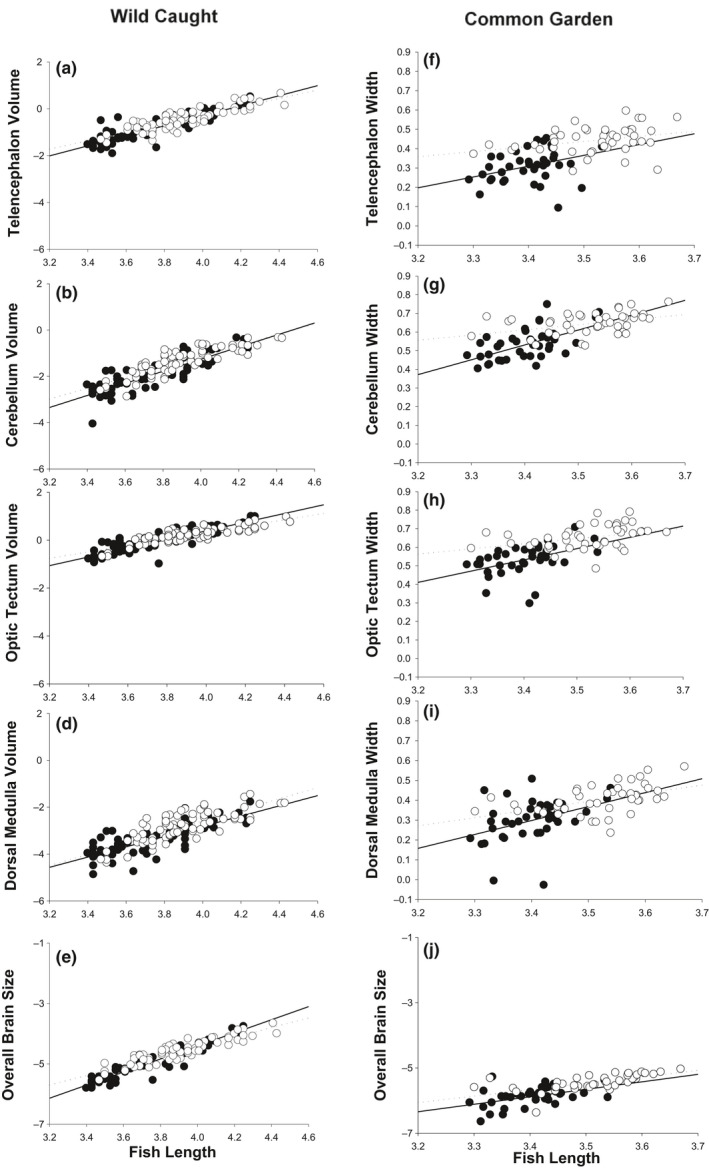
Regressions between fish length (*x*‐axis) and overall brain size or brain structure for wild caught fish and common garden‐reared fish (all data was ln‐transformed). The left column shows wild‐caught fish (panels a–e), and the right column shows common garden‐reared fish correlations (f–j). RO data are represented by open circles and the dotted line, and the HP population is represented by the solid circles and solid line

We observed a significant (*p* < .05) ‘river × predation × length’ interaction for the volume of all measured brain structures (dorsal medulla, cerebellum, optic tectum, and telencephalon; Table 1), due to the differing allometries between total length and brain size or structure across rivers and populations (Figure 1, Figure S2). We also observed a significant ‘river x predation’ interaction for the volume of all measured brain structures (Table 1). These significant interactions were due to contrasting patterns of divergence in brain structures between HP and RO sites among the focal rivers. For example, RO fish from the Arima River exhibited a 17% larger dorsal medulla volume compared with Arima HP fish while the differences in dorsal medulla volume were smaller (Guanapo) or in the opposite direction (Aripo) in the other rivers (Figure S2). Overall, there was a significant (*p* < .05) difference in the volume of the optic tectum but not the dorsal medulla, cerebellum, or telencephalon between HP and RO sites (Table 1, Figure 1). The volume of the optic tectum was 23% greater in RO fish than HP fish (Figure 1). Although the differences were nonsignificant, RO fish qualitatively exhibited a larger dorsal medulla, cerebellum, and telencephalon compared with HP fish (Figure 1).

**Table 1:** Results of linear mixed models for brain regions of wild‐caught fish with ln‐length included as a covariate

Brain region	Predictor	*df*	*F*	*p*‐value
Telencephalon	River	2	2.5050	.086
	Predation	1	0.806	.371
	**River × predation**	2	11.046	.000
	River × length	2	2.418	.093
	**River × predation × length**	3	9.065	.000
Dorsal Medulla	River	2	1.557	.215
	Predation	1	0.014	.907
	**River × predation**	2	5.925	.003
	**River × predation × length**	5	2.648	.026
Optic Tectum	River	2	1.559	.214
	**Predation**	1	6.349	.013
	**River × predation**	2	14.896	.000
	**Predation × length**	1	5.787	.018
	**River × predation × length**	4	9.232	.000
Cerebellum	**River**	2	3.904	.023
	Predation	1	1.975	.162
	**River × predation**	2	8.392	.000
	**River × length**	2	3.518	.032
	**River × predation × length**	3	7.425	.000

#### 3.1.2 Common garden

We observed significant (*p* < .05) differences in the width of all four brain structures between the focal populations (optic tectum, telencephalon, dorsal medulla, and cerebellum; Table 2, Figure 1). RO fish exhibited larger brain regions than HP fish for all measured structures (Figure 1). The width of the optic tectum, telencephalon, dorsal medulla, and cerebellum was 14%, 22%, 21%, and 11% greater in RO fish than HP fish, respectively. The ‘river × predation’ and ‘river × predation × length’ interactions were not significant and not included in the final model for any of the brain structures (Figure 1; Figure S3). The contrasting food treatments were not significant (Table 2). All ‘predation × food’ interactions were not significant and not included in the final models (*F*‐value < 1.0), but the ‘predation × food × length’ interaction was significant for the cerebellum (Table 2).

**Table 2:** Results of linear mixed models for brain regions of common garden‐reared fish with ln‐length included as a covariate

Brain region	Predictor	*df*	*F*	*p*‐value
Telencephalon	River	1	1.581	.212
	**Predation**	1	10.144	.002
	Food	1	2.314	.132
	River × length	1	1.616	.207
Dorsal Medulla	River	1	0.088	.768
	**Predation**	1	4.667	.034
	Food	1	3.312	.073
Optic Tectum	River	1	0.233	.631
	**Predation**	1	9.796	.002
	Food	1	1.804	.183
Cerebellum	River	1	1.123	.293
	**Predation**	1	11.647	.001
	Food	1	0.128	.722
	**Food × predation × length**	3	3.920	.012

### 3.2 Covariation between brain size and eye size

#### 3.2.1 Wild caught

**Figure 2:**
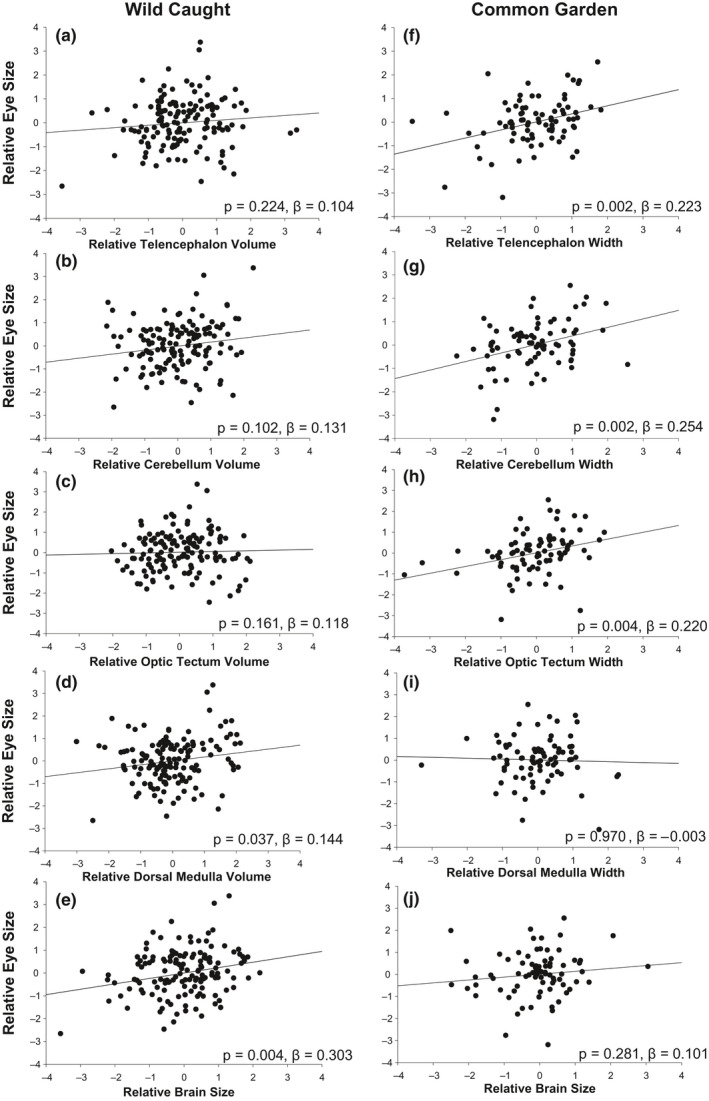
Regressions between relative brain region or relative brain size, and relative eye size. Relative brain structures were generated by outputting the residuals from regressions between an individual ln‐brain structure versus ln‐fish length. Relative brain size and relative eye size were generated by outputting residuals from regressions between ln‐eye or ln‐brain size and ln‐fish length. The left column represents the wild caught fish (panels a–e), and the right column shows the common garden‐reared fish data (f–j)

The results of a multiple regression revealed a significant (*p* < .05) link between overall brain size and eye size and dorsal medulla and eye size (Figure 2, Table S4). When separating the multiple regressions by population, we observed that the connection between the dorsal medulla and relative eye size was significant for RO fish (*p* = .006; Figure S4). All other connections between brain structures and eye size stemming from the multiple regressions were not significant. None of the ‘predation × brain structure’ interactions from the GLMs were significant.

#### 3.2.2 Common garden

The multiple regressions revealed a significant positive relationship between the size of the telencephalon, optic tectum, cerebellum, and eye size (Figure 2, Table S4). The connections between brain size and eye size and dorsal medulla and eye size stemming from the multiple regressions were not significant (Figure 2). When separating the multiple regressions by population, none of the links between brain size or structure and eye size were significant (Figure S5). None of the ‘predation × brain structure’ interactions were significant.

## 4 DISCUSSION

The evolution of larger brains is associated with shifts in brain architecture in Trinidadian killifish. Fish stemming from common garden experiments revealed that killifish from RO sites have evolved a wider (based upon diameter) telencephalon, optic tectum, cerebellum, and dorsal medulla than fish from HP sites (Figure 1, Figure S3). These patterns were similar in wild‐caught specimen but were larger in magnitude and more consistent in common garden‐reared fish. In the wild‐caught fish, the overall differences between HP and RO sites and the allometric relationships between brain structure and eye size often varied across rivers. Yet, the consistent pattern of divergence between RO and HP fish observed in the second‐generation common garden‐reared fish indicates such differences are likely genetic in origin (Figure 1). We also observed a significant connection between brain and eye size; in the common garden‐reared fish we found a positive correlation between the size of the telencephalon, optic tectum, cerebellum, but not overall brain size, and eye size (Figure 2). This provides evidence for evolutionary covariation between the components of the brain and eye size. However, it is important to note that we failed to observe a consistent pattern between the wild‐caught and common garden‐reared specimens and that we cannot rule out the influence of grandparental or transgenerational effects on the differences in brain regions for common garden‐reared fish. Collectively, our data indicate that phenotype (wild caught) does not necessarily predict genotype (common garden) in brain structure or in the connection between brain and eye size.

What ecological features might lead to the contrasting patterns of brain architecture evolution observed between wild‐caught and common garden‐reared fish? As described in the introduction, it is important to note that differences in predation are associated with correlated shifts in ecological factors that may influence the expression of brain size, brain structure, and eye size. Increased rates of predation in high predation sites is correlated with increased light and resource availability (El‐Sabaawi et al., 2012; Grether et al., 2001; Reznick, Butler IV, & Rodd, 2001). Killifish are known to mostly forage on invertebrates (Fraser et al., 1999), and invertebrate abundance varies with predation intensity (El‐Sabaawi et al., 2012). Invertebrate abundance is 40%–173% greater in HP sites than RO sites (El‐Sabaawi et al., 2012). Furthermore, canopy cover is 5%–27% more open in HP sites than RO sites in the same rivers used in this study (El‐Sabaawi et al., 2012). These differences in canopy cover and resource availability are also known to vary across streams. For example, the Guanapo RO sites have 7% more open canopy than Arima and Aripo RO sites, while invertebrate abundance is 80% higher in Guanapo RO sites compared with the Aripo RO (El‐Sabaawi et al., 2012). These differences across streams may alter the expression of traits at the phenotypic level. We explored potential connections between the published estimates of light and food availability (El‐Sabaawi et al., 2012) and the brain and eye size values in the current study via multiple regressions. Such exploratory analyses did not yield significant connections between the environmental data and the neurosensory traits of killifish (Table S5).

The disconnection between our wild‐caught specimens and common garden‐reared samples is not necessarily surprising because a growing number of studies have shown that brain size (and brain components) is plastic and sensitive to changes in the environment. For example, brain mass was shown to be plastic across and within populations of an African cichlid (*Pseudocrenilabrus multicolor victoriae*) that experience differing oxygen levels (Crispo & Chapman, 2010). Differences in the size and plasticity of brain size, optic tectum, and olfactory bulbs were documented in stickleback from marine and pond habitats that were reared in differing social environments (Gonda et al., 2009a, 2009b). Another study in extremophile fish (*Poecilia Mexicana*) found plasticity in brain region volumes (i.e., cerebellum, optic tectum, telencephalon) across varying levels of light and sulfide exposure (Eifert et al., 2015). Competition and predation were shown to induce plastic responses in brain size and structures (i.e., optic tectum, medulla oblongata) in tadpoles (Gonda et al., 2010). This work collectively signals that a multitude of ecological variables likely influenced the patterns of variation in the components of the brain in the wild‐caught specimens.

One surprising aspect of our results is that we did not find a connection between overall brain size and eye size in common garden‐reared fish. However, we did find connections between eye size and three brain structures—telencephalon, optic tectum, and cerebellum. We expected to see positive relationships between eye size and the telencephalon and optic tectum, but not necessarily between eye size and the cerebellum. However, the connection between eye size and cerebellum is potentially intuitive given that the cerebellum is involved in coordinating motor activity (Broglio et al., 2003) and more specifically may play a role in spatial awareness, spatial orientation, and eye movement (Kotrschal et al., 1998). The cerebellum may also be indirectly correlated with eye size via connections to the telencephalon and optic tectum. Studies have indeed shown positive associations between cerebellum size and optic tectum, telencephalon size (Huber & Rylander, 1992; Van Staaden et al., 1995), suggesting a functional unit upon which selection acts upon in similar ways. These results suggest that selection (directly or indirectly) acts upon specific regions of the brain, and not overall brain size, to enhance visual capabilities. Our results also expand current understanding of the connection between eye and brain size by identifying links between eye size and specific brain structures. Our results now call for more mechanistic studies that specifically quantify the functional implications of variation in brain architecture and eye size and their potential connections with visual acuity and behavioral aspects of visual performance.

There are multiple plausible adaptive explanations for the observed differences in brain structure between RO and HP killifish and the observed connection between brain architecture and eye size. Previous work has suggested that competition for food and mates requires high cognitive abilities (Barkley & Jacobs, 2007; Jacobs, 1996; Jacobs et al., 1990). This is important because RO sites are characterized by high killifish density, low resources, and presumably intense competition for both food and mates compared with fish in HP sites (Fraser et al., 1999; Gilliam et al., 1993). Thus, larger brain structures that help overcome these obstacles are likely favored in RO sites. That is, the evolution of larger brain structures that are connected to learning, movement, and coordination may be driven by selection for increased investment in foraging and mating capabilities that enhance fitness in high competition environments. For example, cognitive function is controlled by the telencephalon (Bshary et al., 2002; Vargas et al., 2009) and the telencephalon is also associated with learning and spatial behaviors such as foraging and mating (Broglio et al., 2003; Portavella et al., 2002). Male killifish can maximize their reproductive fitness by mating with as many females as possible, which likely requires them to move frequently throughout the stream. Research has indeed shown that males exhibit greater movement than females in natural streams (K. J. Howell & M. R. Walsh, unpublished data). Thus, an increase in the size of the telencephalon may enhance spatial memory and mating opportunities and therefore reproductive fitness. The connection between brain structures and eye size is also potentially adaptive. Previous work in killifish has shown that increases in eye size are associated with greater survival and enhanced growth in sites that lack predators (Beston & Walsh, 2019). This suggests that the increased competition for food in RO sites selects for increased eye size and that brain structures may evolve as an indirect byproduct. Experimental tests are now needed to determine the fitness advantages of the connection between brain and eye size.

## 5 CONCLUSIONS

Here, we found that decreased predation was associated with the evolution of larger components of the brain in fish that were reared for multiple generations in the laboratory (Figure 1). These same data also revealed positive correlations between brain components (but not brain size) and eye size, providing evidence for covariation between brain architecture and eye size (Figure 2). However, inconsistent patterns between wild‐caught and common garden‐reared fish highlight that varying ecological conditions across streams may alter phenotypic expression of traits. Overall, our results provide support for covariation in brain component and eye size evolution, and we propose that selection, directly or indirectly, acts upon specific brain regions rather than overall brain size to increase visual system function. More tests are now needed to understand the fitness and functional advantages of selection for larger eyes, brains, and brain structures.

## CONFLICT OF INTEREST

None declared.

## AUTHOR CONTRIBUTIONS

**Kaitlyn J. Howell:** Formal analysis (equal); investigation (equal); methodology (equal); writing–original draft (equal); writing–review and editing (equal). **Shannon M. Beston:** Data curation (equal). **Sara Stearns:** Data curation (equal). **Matthew R. Walsh:** Formal analysis (equal); investigation (equal); methodology (equal); writing–original draft (equal); writing–review and editing (equal).

## Supporting information

Supplementary Material

## Data Availability

All data have been made available on Dryad. https://doi.org/10.5061/dryad.2547d7wpd.
