# Supplementary material for: Coordinated evolution of brain size, structure, and eye size in Trinidadian killifish
Source: Ecol Evol. 2020 Nov 22;11(1):365–75. doi: 10.1002/ece3.7051 (PMC7790632; doi:10.1002/ece3.7051)
Supplement: Supplementary file 1 — Supplementary Material [file ECE3-11-365-s001.docx]

**Appendix A.** Supporting Tables and Figures

**Supplemental Table 1**. Results from linear mixed models for brain regions of wild caught fish with ln-brain size as the covariate.

| Brain Region | Predictor | df | F | p-value |
| --- | --- | --- | --- | --- |
| Telencephalon | River | 2 | 0.539 | 0.585 |
|  | **Predation** | 1 | 4.506 | 0.036 |
|  | **River x predation** | 2 | 8.477 | 0.000 |
|  | Predation x brain | 1 | 3.930 | 0.050 |
|  | **River x predation x brain** | 4 | 5.953 | 0.000 |
| Dorsal Medulla | River | 2 | 0.267 | 0.766 |
|  | Predation | 1 | 2.888 | 0.092 |
|  | Predation x brain | 1 | 2.112 | 0.149 |
|  | **River x predation x brain** | 4 | 2.867 | 0.026 |
| Optic Tectum | River | 2 | 0.498 | 0.609 |
|  | Predation | 1 | 0.128 | 0.721 |
|  | **River x predation** | 2 | 6.266 | 0.003 |
|  | **River x predation x brain** | 5 | 4.017 | 0.002 |
| Cerebellum | River | 2 | 1.532 | 0.220 |
|  | Predation | 1 | 0.433 | 0.512 |
|  | **River x predation** | 2 | 5.216 | 0.007 |
|  | River x brain | 2 | 1.620 | 0.202 |
|  | **River x predation x brain** | 3 | 4.348 | 0.006 |

**Supplemental Table 2.** Correlations between the width and volume of each brain structure (wild caught data only).

|  | Telencephalon | Dorsal Medulla | Optic Tectum | Cerebellum |
| --- | --- | --- | --- | --- |
| Pearson correlation (r) | 0.407 | 0.416 | 0.456 | 0.309 |
| p-value | 0.000 | 0.000 | 0.000 | 0.000 |

**Supplemental Table 3**. Results of linear mixed models for brain regions of common garden fish with ln-brain size as the covariate.

| Brain Region | Predictor | df | F | p-value |
| --- | --- | --- | --- | --- |
| Telencephalon | River | 1 | 0.003 | 0.954 |
|  | **Predation** | 1 | 8.503 | 0.005 |
|  | Food | 1 | 0.832 | 0.364 |
|  | River x Food | 1 | 1.550 | 0.217 |
| Dorsal Medulla | River | 1 | 1.201 | 0.277 |
|  | Predation | 1 | 3.664 | 0.059 |
|  | Food | 1 | 0.327 | 0.569 |
|  | Food x predation | 1 | 2.859 | 0.095 |
|  | Predation x brain | 1 | 3.204 | 0.078 |
|  | **River x predation x brain** | 2 | 4.421 | 0.015 |
|  | Food x predation x brain | 2 | 1.475 | 0.235 |
| Optic Tectum | River | 1 | 1.992 | 0.162 |
|  | **Predation** | 1 | 14.019 | 0.000 |
|  | Food | 1 | 0.065 | 0.800 |
| Cerebellum | **River** | 1 | 4.043 | 0.048 |
|  | Predation | 1 | 0.230 | 0.633 |
|  | Food | 1 | 0.100 | 0.753 |
|  | **River x predation** | 1 | 5.636 | 0.020 |
|  | River x brain | 1 | 3.555 | 0.063 |
|  | River x predation x brain | 2 | 2.792 | 0.068 |

**Supplemental Table 4.** Multiple regressions between brain size or structure and eye size with ln-length included as a covariate for both wild caught and common garden specimens. The r-square values and overall p-values represent the predictive ability of the full model. Significant p-values for brain structures are bolded.

|  |  | Wild-Caught | | | |  | Common Garden | | | | |
| --- | --- | --- | --- | --- | --- | --- | --- | --- | --- | --- | --- |
| **Brain Region** | **R square** | **Overall p** | **t** | **β** | **p-value** | **R square** | | **Overall p** | **t** | **β** | **p-value** |
| Telencephalon | 0.774 | 0.000 | 1.222 | 0.104 | 0.224 | 0.771 | | 0.000 | 3.917 | 0.223 | **0.002** |
| Ln Length |  |  | 9.212 | 0.786 | 0.000 |  |  |  | 10.354 | 0.721 | 0.000 |
| Dorsal Medulla | 0.779 | 0.000 | 2.103 | 0.144 | **0.037** | 0.741 | | 0.000 | -0.038 | -0.003 | 0.970 |
| Ln Length |  |  | 11.125 | 0.761 | 0.000 |  |  |  | 11.866 | 0.862 | 0.000 |
| Optic Tectum | 0.775 | 0.000 | 1.410 | 0.118 | 0.161 | 0.768 | | 0.000 | 3.011 | 0.220 | **0.004** |
| Ln Length |  |  | 9.245 | 0.775 | 0.000 |  |  |  | 9.770 | 0.714 | 0.000 |
| Cerebellum | 0.776 | 0.000 | 1.645 | 0.131 | 0.102 | 0.771 | | 0.000 | 3.249 | 0.254 | **0.002** |
| Ln Length |  |  | 9.631 | 0.766 | 0.000 |  |  |  | 8.687 | 0.678 | 0.000 |
| Overall Brain | 0.785 | 0.000 | 2.889 | 0.303 | **0.004** | 0.731 | | 0.000 | 1.085 | 0.101 | 0.281 |
| Ln Length |  |  | 5.696 | 0.597 | 0.000 |  |  |  | 8.296 | 0.775 | 0.000 |

**Supplemental Table 5.** Regressions between environmental factors, brain size and structure, and eye size. Environmental data is an average from multiple samples at each site (see El-Sabaawi et al. 2012), therefore all eye and brain data were averaged from ln-transformed data in order to run multiple regression.

|  | Environmental Factor | β | t | p-value |
| --- | --- | --- | --- | --- |
| Telencephalon | Algae Abundance | -0.369 | -1.748 | 0.331 |
|  | Invertebrate Abundance | -1.184 | -3.744 | 0.166 |
|  | Light (% Canopy) | -0.032 | -0.128 | 0.919 |
| Dorsal Medulla | Algae Abundance | 0.247 | 1.014 | 0.496 |
|  | Invertebrate Abundance | -1.479 | -4.052 | 0.154 |
|  | Light (% Canopy) | 0.348 | 1.211 | 0.439 |
| Optic Tectum | Algae Abundance | -0.531 | -0.481 | 0.714 |
|  | Invertebrate Abundance | 0.169 | 0.102 | 0.935 |
|  | Light (% Canopy) | 0.220 | 0.170 | 0.893 |
| Cerebellum | Algae Abundance | -0.051 | -0.135 | 0.915 |
|  | Invertebrate Abundance | -1.336 | -2.365 | 0.255 |
|  | Light (% Canopy) | 0.111 | 0.248 | 0.307 |
| Overall Brain | Algae Abundance | -0.014 | -0.202 | 0.873 |
|  | Invertebrate Abundance | -0.294 | -3.800 | 0.164 |
|  | Light (% Canopy) | -0.169 | -2.311 | 0.260 |
| Eye | Algae Abundance | 0.057 | 0.207 | 0.870 |
|  | Invertebrate Abundance | 0.000 | 0.000 | 1.000 |
|  | Light (% Canopy) | -0.465 | -1.632 | 0.350 |


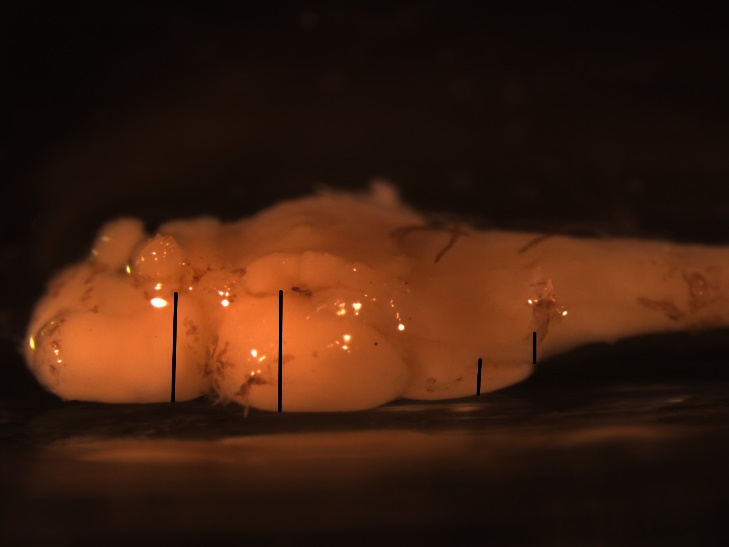

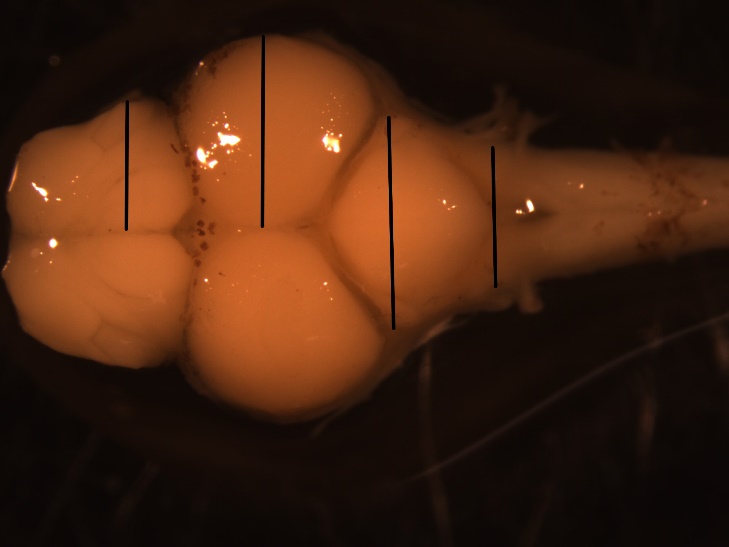

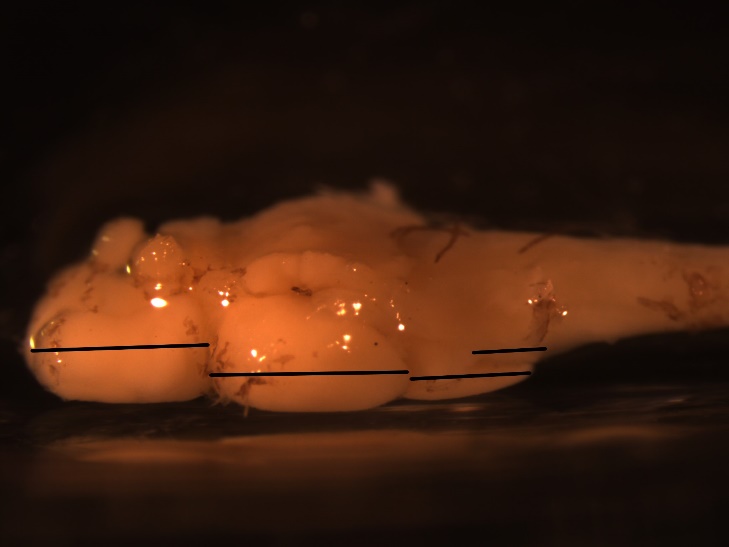


B.

1.

2.

3.

4.

A.

1.

2.

3.

4.

C.

1.

2.

3.

4.

**Supplemental Figure 1.** A) Dorsal view of the brain with width measurements B) Lateral view of height measurements C) Lateral view of length measurements. Brain components are represented by numbers: (1) telencephalon, (2) optic tectum, (3) cerebellum, (4) dorsal medulla.

HP

RO

RO

HP

HP

RO

**A.**

**B.**

**C.**

**D.**

**E.**

**F.**

**G..**

**H..**

**I..**

**J..**

**K..**

**L..**

**M.**

**N.**

**O.**

**Wild Caught - Arima**

**Wild Caught - Aripo**

**Wild Caught - Guanapo**

**Supplemental Figure 2.** Regressions of fish length (x-axis) and overall brain size or brain structure volume separated by river for wild caught fish. HP populations are represented by the black circles and solid black lines; RO populations are represented by the open circles and the dashed lines.

**Common Garden – Arima**

**Common Garden – Guanapo**

**A.**

**B.**

**C.**

**D.**

**E.**

**F.**

**G..**

**H.**

**I..**

**J..**

RO

HP

RO

HP

**Supplemental Figure 3.** Regressions between fish length (x-axis) and overall brain size or brain structure width separated by river and predation for wild caught fish. Arima River is the left column (panels A-E), and Guanapo River is the right column (panels F-J). HP populations are represented by the solid circles and solid black lines; RO populations are represented by the open circles and the dashed lines.

p= 0.575, β=0.070, t=0.563

p= 0.188, β=0.163, t=1.330

p= 0.486, β=0.090, t=0.700

p= 0.006, β=0.282, t=2.826

p= 0.277, β=0.159, t=1.096

p= 0.939, β=0.009, t=0.077

p= 0.664, β= -0.045, t= -0.436

p= 0.454, β=0.087, t=0.753

p= 0.359, β= -0.083, t= -0.924

p= 0.183, β=0.230, t=1.346

**A.**

**B.**

**C.**

**D.**

**E.**

**F.**

**G..**

**H..**

**I..**

**J..**

**Wild Caught RO** HP

**Wild Caught HP** HP

**Supplemental Figure 4.** Regressions between relative brain region, relative brain size, and relative eye size by population for wild caught fish. The left column represents RO fish (panels A-E), and the right column shows HP fish (F-J).

**A.**

**B.**

**C.**

**D.**

**E.**

**F.**

**G..**

**H..**

**I.**

**J..**

**Common Garden HP** HP

**Common Garden RO** HP

p= 0.761, β=0.034, t=0.306

p= 0.682, β=0.132, t=1.124

p= 0.452, β=0.087, t=0.759

p= 0.402, β= -0.103, t= -0.846

p= 0.535, β= -0.100, t= -0.626

p= 0.150, β=0.176, t=1.473

p= 0.266, β=0.156, t=1.131

p= 0.272, β=0.139, t=1.116

p= 0.508, β= -0.082, t= -0.669

p= 0.917, β=0.014, t=0.105

**Supplemental Figure 5.** Regressions between relative brain region or relative brain size, and relative eye size for common garden fish. The left column represents RO fish (panels A-E), and the right column shows HP fish (F-J).
